# Whole-genome mapping of quantitative trait loci and accuracy of genomic predictions for resistance to columnaris disease in two rainbow trout breeding populations

**DOI:** 10.1186/s12711-019-0484-4

**Published:** 2019-08-06

**Authors:** Rafael M. O. Silva, Jason P. Evenhuis, Roger L. Vallejo, Guangtu Gao, Kyle E. Martin, Tim D. Leeds, Yniv Palti, Daniela A. L. Lourenco

**Affiliations:** 1 National Center for Cool and Cold Water Aquaculture, Agricultural Research Service, United States Department of Agriculture, 11861 Leetown Road, Leetown, WV 25430 USA; 2 Department of Animal and Dairy Science, University of Georgia, Athens, 425 River Road, Athens, GA 30602 USA; 3 Present Address: Zoetis, Sao Paulo, Sao Paulo, 04711-130 Brazil; 4 Troutloged, Inc., P.O. Box 1290, Sumner, WA 98390 USA

## Abstract

**Background:**

Columnaris disease (CD) is an emerging problem for the rainbow trout aquaculture industry in the US. The objectives of this study were to: (1) identify common genomic regions that explain a large proportion of the additive genetic variance for resistance to CD in two rainbow trout (*Oncorhynchus mykiss*) populations; and (2) estimate the gains in prediction accuracy when genomic information is used to evaluate the genetic potential of survival to columnaris infection in each population.

**Methods:**

Two aquaculture populations were investigated: the National Center for Cool and Cold Water Aquaculture (NCCCWA) odd-year line and the Troutlodge, Inc., May odd-year (TLUM) nucleus breeding population. Fish that survived to 21 days post-immersion challenge were recorded as resistant. Single nucleotide polymorphism (SNP) genotypes were available for 1185 and 1137 fish from NCCCWA and TLUM, respectively. SNP effects and variances were estimated using the weighted single-step genomic best linear unbiased prediction (BLUP) for genome-wide association. Genomic regions that explained more than 1% of the additive genetic variance were considered to be associated with resistance to CD. Predictive ability was calculated in a fivefold cross-validation scheme and using a linear regression method.

**Results:**

Validation on adjusted phenotypes provided a prediction accuracy close to zero, due to the binary nature of the trait. Using breeding values computed from the complete data as benchmark improved prediction accuracy of genomic models by about 40% compared to the pedigree-based BLUP. Fourteen windows located on six chromosomes were associated with resistance to CD in the NCCCWA population, of which two windows on chromosome Omy 17 jointly explained more than 10% of the additive genetic variance. Twenty-six windows located on 13 chromosomes were associated with resistance to CD in the TLUM population. Only four associated genomic regions overlapped with quantitative trait loci (QTL) between both populations.

**Conclusions:**

Our results suggest that genome-wide selection for resistance to CD in rainbow trout has greater potential than selection for a few target genomic regions that were found to be associated to resistance to CD due to the polygenic architecture of this trait, and because the QTL associated with resistance to CD are not sufficiently informative for selection decisions across populations.

**Electronic supplementary material:**

The online version of this article (10.1186/s12711-019-0484-4) contains supplementary material, which is available to authorized users.

## Background

Rainbow trout (*Oncorhynchus mykiss*) is an economically important aquaculture commodity. However, diseases have become one of the main constraints to sustainable aquaculture production and trade [1]. Parasites and infectious diseases cause significant losses in aquaculture worldwide, accounting for 90% of the total loss of all trout intended for sale in the United States during 2016 (USDA National Agricultural Statistics Service).

Columnaris disease (CD) is an emerging problem for rainbow trout aquaculture in the US, which is caused by the gram-negative bacterium *Flavobacterium columnare*. CD is distributed around the world and infects cultured and wild freshwater fish species, such as rainbow trout, tilapia and channel catfish. It can infect fish of all ages, but it is more frequent in young fish, and it can cause major losses [2, 3]. Transmission occurs often horizontally and indirectly through the water column without fish-to-fish contact, and generally the severity and occurrence are greater at warmer water temperatures [2].

Currently there are no licensed vaccines against *F. columnare* for use in rainbow trout and regulations on the use of antibiotics in aquaculture are very restrictive. Hence, genetic improvement of resistance to the disease through selective breeding offers a sustainable and long-term alternative approach for this problem. Recently, we have reported that resistance to CD is heritable and positively correlated with resistance to bacterial cold water disease (BCWD), which is caused by *Flavobacterium psychrophilum* [4]. Two aquaculture-relevant rainbow trout breeding populations were used in this study: the odd-year line of the National Center for Cool and Cold Water Aquaculture (NCCCWA; heritability = 0.23 and genetic correlation = 0.40), and the Troutlodge, Inc., May odd-year (TLUM) nucleus breeding population (heritability = 0.34 and genetic correlation = 0.39) [4]. Therefore, there is a great potential to genetically improve resistance to CD in rainbow trout using selective breeding. In addition, recently we showed that genomic selection for resistance to BCWD holds great potential for rapid improvement in the same TLUM population [5]. However, a better understanding of the genetic architecture of this trait is needed to guide decisions on whether to apply genomic tools in selective breeding strategies, and to determine which genomic-assisted breeding approach is likely to be the most successful for this trait in both populations. The genomic-assisted breeding approaches can consider information from all the single nucleotide polymorphisms (SNPs) on a chip (i.e., genome-wide selection) or only from the quantitative trait loci (QTL) that explain a high proportion of the variance (i.e., genome-targeted selection) [6]. In the current study, we added genotype data to a subset of the population (year-class 2015) from the previous study, which included only pedigree and phenotype data [4]. The objectives of this study were: (1) to identify common genomic regions that explain a large proportion of the additive genetic variance for resistance to CD in a single generation of the NCCCWA and TLUM rainbow trout nucleus breeding populations; and (2) to estimate the gains in accuracy of prediction when genomic information is used to evaluate the genetic potential of surviving to columnaris infection in each population.

## Methods

### Data and disease challenge

**Fig. 1:**
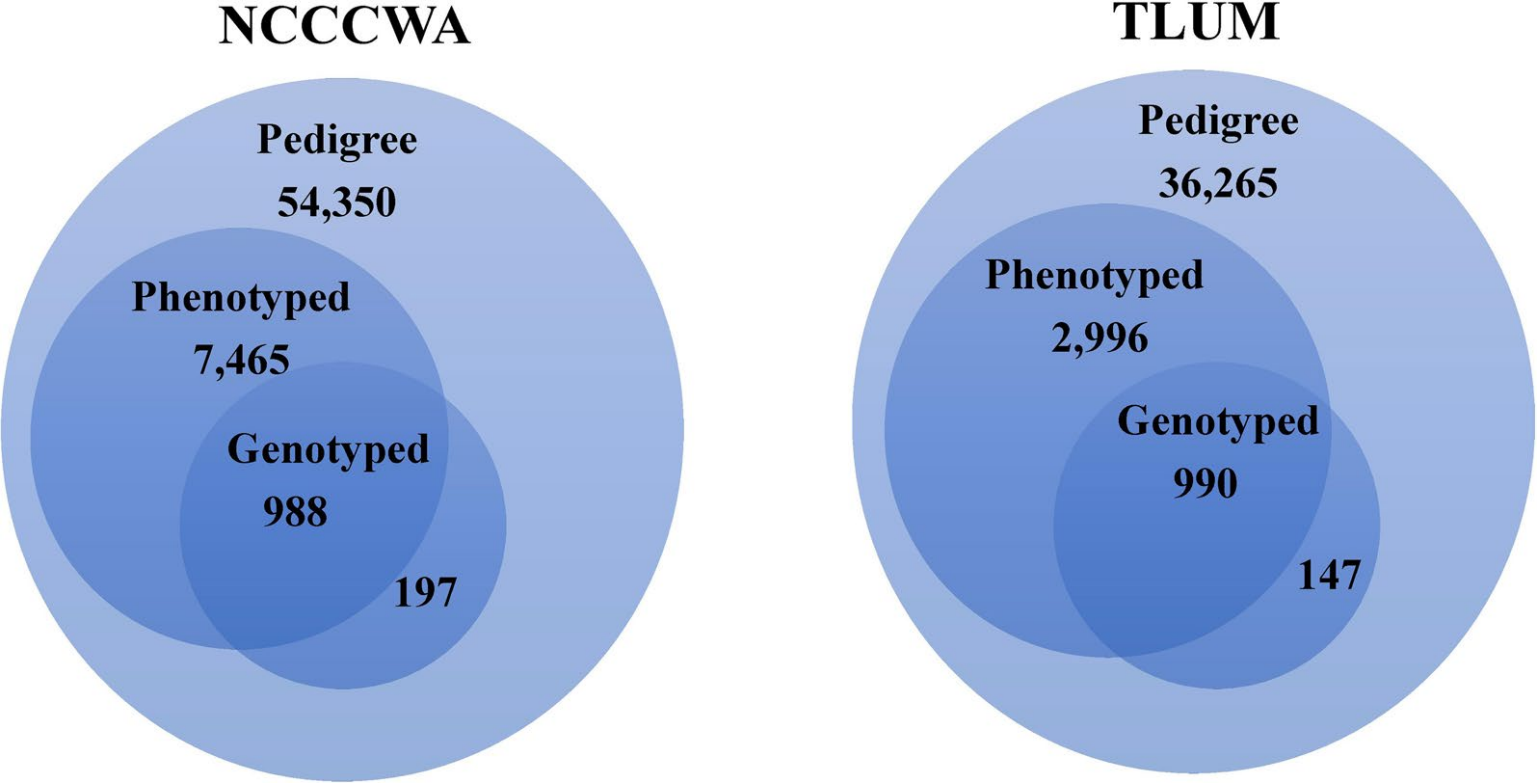
A Venn diagram for the number of fish included in the pedigree, phenotype and genotype datasets for each population. *NCCCWA* National Center for Cool and Cold Water Aquaculture, *TLUM* Troutlodge, Inc. USA May spawning nucleus population

Two important aquaculture populations were investigated: the National Center for Cool and Cold Water Aquaculture (NCCCWA; Leetown, WV) odd-year line (ARS-Fp-R), which has been under family-based selective breeding for improved resistance to BCWD for five generations [7, 8], and the Troutlodge, Inc., May-spawning odd-year (TLUM) nucleus breeding population, which has been subjected to at least six generations of selective breeding for growth-related traits, but is considered unselected for traits related to disease resistance. The TLUM odd-year line is one of eight domestic rainbow trout nucleus populations that are maintained by Troutlodge, Inc. (Sumner, WA), a major rainbow trout breeding company globally. Both populations have been closed to outside germplasm and are scheduled to produce approximately 100 nucleus families at each generation. The sampling structure for each population is illustrated in Fig. 1. The numbers of fish in the pedigree records were 54,350 and 36,265, for the NCCCWA and TLUM populations, respectively, with 8453 and 3986 fish recorded for resistance to CD, respectively. Genotype records with the 57 k SNP array (Affymetrix Axiom^®^) [9] were available for 1185 (197 parents and 988 phenotyped offspring) and 1137 (147 parents and 990 phenotyped offspring) fish from the NCCCWA and TLUM populations, respectively. The sampling scheme for genotyping aimed at including five mortalities and five survivors per family, although fewer survivors or mortalities were available for sampling for some families. After SNP quality control, 35,900 and 33,980 high-quality and informative SNPs were used in the analysis, respectively for the NCCCWA and TLUM populations.

All post-hatch rearing practices and disease challenges were conducted at the USDA, ARS, NCCCWA, as previously described [3]. The NCCCWA fish were spawned in February 2015 and the TLUM in May 2015. Nucleus TLUM families were shipped to the NCCCWA as eyed eggs and reared as separate families following standard NCCCWA culture practices until the time of disease challenge. Each family was challenged in two tank replicates and the challenge counted for 40 and 20 fry per tank, respectively for NCCCWA and TLUM. Pathogen-naïve fry were challenged with CD by immersion during 1 h in a static bath of a virulent strain (CSF298-10) of *F. columnaris*. To ensure an equal dose of bacteria in all tanks, each tank was inoculated with 40 mL of liquid culture (using a graduated pipette) that had a final OD540 of ~ 0.7. Twelve individual tanks were sampled during the bath challenge and plated to quantify colony forming unit (CFU)/mL. No significant differences were found between tanks. The disease survival challenge for the NCCCWA and TLUM fish started at 66 days post-hatch (BW ~ 1.3 g) and 78 days post-hatch (BW ~ 2.8 g), respectively. The target for the immersion challenge dose was 2 × 10^8^ CFU per mL of water, and the actual dose was 1.97 × 10^7^ and 2.08 × 10^8^ CFU for the NCCCWA and TLUM fish, respectively. Fry were reared in heated flow-through spring water (16 °C) for the duration of the 21-day CD challenge period and dead fish were removed and recorded daily for each tank. Fry was categorized as 2 if it survived for the 21-day post challenge period or 1 otherwise. Inspections were conducted to confirm the presence of the target pathogen in dead fish that were collected during the disease survival trial. The Institutional Animal Care and Use Committee (USDA-ARS, Leetown, WV) reviewed and approved all the protocols used for disease challenge.

### Model and analyses

#### Identification of common genomic regions

The status of resistance to CD was considered categorical and modelled using a threshold approach [10]. The model for the underlying liability of resistance to CD was:

$${\mathbf{U}} = {\mathbf{X}}{\varvec{\upbeta }} + {\mathbf{Za}} + {\mathbf{Wf}} + {\mathbf{e}},$$

where ${\mathbf{U}}$ is the vector of the underlying distribution of resistance to CD; ${\varvec{\upbeta}}$ is the vector of systematic effects (system and row); ${\mathbf{a}}$ is the vector of additive direct genetic effects; ${\mathbf{f}}$ is a vector of random family/tank effects; ${\mathbf{e}}$ is the vector of random residuals; ${\mathbf{X}}$, ${\mathbf{Z}}$, and ${\mathbf{W}}$ are the incidence matrices for the effects contained in **β**, **a**, and **f**, respectively. It was assumed that the additive direct genetic, family/tank, and residual effects were normally distributed with a mean equal to 0 and variances given by $var\left( {\mathbf{a}} \right) = {\mathbf{K}}\sigma_{\text{a}}^{2}$, $var\left( f \right) = {\mathbf{I}}\sigma_{f}^{2}$, and $var\left( e \right) = {\mathbf{I}}\sigma_{e}^{2}$, respectively, where ${\mathbf{K}}$ is ${\mathbf{A}}$, the pedigree relationship matrix, in the best linear unbiased prediction (BLUP) and ${\mathbf{H}}$, the realized relationship matrix, in single-step genomic BLUP (ssGBLUP); ${\mathbf{I}}$ is an identity matrix; $\sigma_{\text{a}}^{2}$, $\sigma_{f}^{2}$, and $\sigma_{e}^{2}$ are the additive direct genetic, family/tank, and residual variances, respectively.

The CD resistance status (y) was modelled with the following distribution:

$$f\left( {y |U} \right) = \mathop \prod \limits_{i = 1}^{n} I\left( {U_{i} < t_{1} } \right)I\left( {y_{i} = 1} \right) + I\left( {U_{i} > t_{1} } \right)I\left( {y_{i} = 2} \right),$$

where $n$ is the number of records, $t_{1}$ the threshold that defines the two survival categories and $I$ is an indicator function that takes value 1 if the fish died during the disease challenge, and 2 if it survived. The procedure is a nonlinear transformation of best linear unbiased estimation (BLUE) and BLUP. Variance components were obtained using the Gibbs sampler applied to threshold models as implemented in THRGIBBSF90 [11].

For the association analyses, we used the weighted single-step genomic BLUP approach for genome-wide association (wssGBLUP) [12]. This method is based on ssGBLUP [13], which has been widely adopted for genomic selection in livestock and aquaculture. Single-step GBLUP combines pedigree and genomic relationships in a single realized relationship matrix (i.e., ${\mathbf{H}}$), and the inverse of ${\mathbf{H}}$ replaces the inverse of the pedigree-based relationship matrix (${\mathbf{A}}^{ - 1}$) in the BLUP mixed model equations.

SNP effects and variances were calculated using wssGBLUP, in an iterative process consisting of seven steps [12]:

1. Set $${\mathbf{D}} = {\mathbf{I}}$$;
2. Construct the genomic relationship matrix ${\mathbf{G}}$ as $\frac{\mathbf{MDM'}}{{ 2\sum {\text{p}}_{\text{j}} ( 1- {\text{p}}_{\text{j}} )}}$;
3. Calculate genomic EBV (GEBV);
4. Calculate the SNP effect: $\widehat{{\mathbf{u}}} = \lambda {\mathbf{DM^{\prime}G}}^{ - 1} \widehat{{\mathbf{a}}}_{g}$, where $\lambda$ is the ratio of SNP variance to additive genetic variances, ${\mathbf{D}}$ is a diagonal matrix of SNP weights, ${\mathbf{M}}$ is a matrix that relates animals to genotypes at each locus, and $\widehat{{\mathbf{a}}}_{g}$ is the GEBV for genotyped animals;
5. Calculate the weight of each SNP: ${\text{d}}_{i} = \widehat{{\mathbf{u}}}_{i}^{2} 2{\text{p}}_{i} \left( {1 - {\text{p}}_{i} } \right)$, where ${\text{p}}$ is the minor allele frequency of the $i$th SNP;
6. Normalize ${\mathbf{D}}$ to keep a constant additive genetic variance;
7. Update $${\mathbf{D}}$$;
8. Iterate from step 2. Only three iterations were used in this study, and the GWAS results from iteration 2, which had the best predictive ability, are presented. As suggested by Wang et al. [12], the iteration with the best predictive ability or greatest accuracy would be the best one to use for GWAS.

Results were summarized using 1-Mb sliding SNP windows. Selection of SNP windows was guided by mapping the genome sequences flanking the SNPs [9] to the new annotated rainbow trout reference genome assembly [14] (GenBank assembly Accession GCA_002163495). The percentage of genetic variance explained by the $i$th window was calculated as described by Wang et al. [15]:

$$\frac{{{\text{var}}\left( {{\text{a}}_{i} } \right)}}{{\sigma_{\text{a}}^{2} }} \times 100{{\% }} = \frac{{{\text{var}}\left( {\mathop \sum \nolimits_{1Mb} {\text{M}}_{j} {\bar{\text{u}}}_{j} } \right)}}{{\sigma_{\text{a}}^{2} }} \times 100{{\% ,}}$$

where ${\text{a}}_{i}$ is the additive genetic effect for the $i$th window.

Genomic regions that explained more than 1% of the additive genetic variance were considered to be associated with resistance to CD.

The software THRGIBBS1F90 via the Gibbs sampler [11] was used to estimate GEBV, whereas POSTGSF90 [11] was used to estimate SNP effects and variances.

#### Evaluation of the accuracy of traditional and genomic predictions

The ability to predict future performance (predictive ability) was calculated using a fivefold cross-validation scheme with five replicates to minimize errors due to single-fold sampling. Predictive ability was calculated for all three iterations of WssGBLUP, providing GEBV1, GEBV2, and GEBV3 for iterations 1, 2, and 3, respectively. Usually when WssGBLUP is used, predictive ability is reported for all iterations. If SNP weighting is beneficial, predictive ability is expected to increase over iterations [12]. For the NCCCWA population, 988 genotyped fish with phenotypes for resistance to CD were available, therefore, fold sampling considered only those fish. For the three- and two-fold sampling, phenotypes were randomly removed for 198 and 197 fish, respectively. For the TLUM population, 990 genotyped fish for resistance to CD were available, and each one of the five folds had phenotypes randomly removed for 198 fish. When phenotypes were removed for one fold (i.e., the validation group), the resulting EBV or GEBV were termed as partial. In each cross-validation fold and replication, posterior means and standard deviation were calculated based on 10,000 Gibbs samples (e.g., no burn-in and all samples were used).

The main interest in salmonid fish breeding is to predict more accurately future performance of young animals; therefore, validation is usually performed on young animals (i.e., forward prediction). However, in our study, most of the genotyped animals were from the same generation, precluding the use of forward prediction. The benchmarks for investigating the ability to predict fish performance were either the phenotypes adjusted for all fixed effects in the model (${\text{y}} - {\text{Xb}}$) or breeding values using complete data (i.e., using data from all five folds). Although the k-fold cross-validation may provide optimistic results because sibs coexist in training and validation populations, this coexistence reflects what happens in real trout breeding programs.

When the benchmark was phenotype adjusted for fixed effects (${\text{y}} - {\text{Xb}}$), the predictive ability (${\text{r}}$) was calculated as the correlation between: ${\text{y}} - {\text{Xb}}$ and $\left( {\text{G}} \right){\text{EBVp}}$, where $\left( {\text{G}} \right){\text{EBVp}}$ is partial EBV or GEBV. Some authors have already shown predictive ability for binary traits (e.g., disease resistance) as the correlation between adjusted phenotypes and EBV or GEBV [16, 17]; however, in such cases the phenotypes are on the observed binary scale and the EBV and GEBV are on the liability scale, making comparisons difficult and results nonsensical. To avoid such problems, we used an alternative method to evaluate the predictive ability of genomic models, which was described by Legarra and Reverter [18] as the LR method. This method compares complete EBV (EBVc) or GEBV (GEBVc), when all data are available, with partial EBV (EBVp) or GEBV (GEBVp), when phenotypes for selection candidates are removed for each fold. This LR method is suitable for models with binary or categorical traits, maternal models, and traits with a low heritability [18]. If the comparison is EBVc with EBVp and GEBVc with GEBVp, we can see how much the breeding values change in the traditional and genomic evaluations when data are added for the validation animals. If the correlation between the partial and complete estimates is higher, the change in breeding value is smaller and the partial data predicts the complete data better. Therefore, the correlation between complete and partial data is a function of the accuracy of prediction. It gives the relative increase in accuracy from one evaluation to the next (i.e., consistency between subsequent evaluations).

Legarra and Reverter [18] proved that $\rho_{EBVp,EBVc}$ is a direct estimator of the increase in accuracy from adding data because its expectation is:

$$E\left( {\rho_{EBVp,EBVc} } \right) = \frac{{\sqrt {1 - \frac{{\overline{{PEV_{p} }} - \overline{{PEC_{p} }} }}{{\left( {1 + \bar{F} - 2\bar{f}} \right)\sigma_{u,\infty }^{2} }}} }}{{\sqrt {1 - \frac{{\overline{{PEV_{c} }} - \overline{{PEC_{c} }} }}{{\left( {1 + \bar{F} - 2\bar{f}} \right)\sigma_{u,\infty }^{2} }}} }},$$

where $PEV$ is prediction error variance or a measure of variance of the estimated minus true breeding value, $PEC$ is the prediction error covariance, $\left( {1 + \bar{F} - 2\bar{f}} \right)$ is the reduction in variance in a selected population due to relationships $\sigma_{u,\infty }^{2}$ is the genetic variance at equilibrium in a population under selection, $\bar{F}$ is the average inbreeding, and $2\bar{f}$ is the average relationship between animals. The term $\sqrt {1 - \frac{{\overline{{PEV_{p} }} - \overline{{PEC_{p} }} }}{{\left( {1 + \bar{F} - 2\bar{f}} \right)\sigma_{u,\infty }^{2} }}}$ is similar to the formula presented by Henderson [19] and later by Van Vleck [20] to compute the accuracy of an EBV:

$$Acc\left( {EBV} \right) = \sqrt {1 - \frac{PEV}{{\left( {1 + F} \right)\sigma_{u}^{2} }}},$$

where $F$ is the inbreeding coefficient of the individual and $\sigma_{u}^{2}$ is the additive genetic variance. Therefore, this shows that $\rho_{EBVp,EBVc}$ and $\rho_{GEBVp,GEBVc}$ are functions of accuracies of EBV.

The regression coefficient (${\text{b}}_{1}$) of complete on partial breeding values (i.e., $\left( {\text{G}} \right){\text{EBVc}} = {\text{b}}_{0} + {\text{b}}_{1} \times \left( {\text{G}} \right){\text{EBVp}})$ was used as a measure of inflation. In the same way, when adjusted phenotypes were used as benchmark, the regression coefficient of adjusted phenotype on partial breeding value (i.e., ${\text{y}} - {\text{Xb}} = {\text{b}}_{0} + {\text{b}}_{1} \times \left( {\text{G}} \right){\text{EBVp}}$) was used to investigate inflation. A value of ${\text{b}}_{1}$ close to 1 means EBV or GEBV are not inflated/over-dispersed [21], because a change of one unit in (G)EBV corresponds to a similar change in adjusted phenotypes.

## Results

### Estimated heritability for resistance to CD

The estimated heritabilities for resistance to CD were 0.32 and 0.51 for the NCCCWA and TLUM populations, respectively, which indicate a moderate to high additive genetic component for the trait. These estimates are higher than our previously reported heritabilities of 0.23 and 0.34 in the NCCCWA and TLUM populations, respectively [4]. Those estimates were based on data combined from the 2013 and 2015 year-classes of each population, while the current heritability estimates were calculated using only data from the 2015 year-class and with a simpler model that did not include the year-class effect.

### Identification of common genomic regions affecting resistance

Descriptive statistics of the family-survival phenotypes and the number of genotyped SNPs that were located in each 1-Mb sliding windows are in Table 1. The mean survival (± SD) was 74.9% (± 13.7%) and 38.6% (± 24.1%), in the NCCCWA and TLUM populations, respectively, which clearly indicate a substantially higher survival rate in the NCCCWA CD challenge. The average number of SNPs per window (± SD) was 27.9 (± 6.2) and 22.2 (± 8.9) for the NCCCWA and TLUM populations, respectively, which means that the genome coverage was better for the NCCCWA population. However, given our recent estimate of strong linkage disequilibrium (LD) (*r*^*2*^ ≥ 0.25) that spans on average over 1 Mb across the rainbow trout genome [22], this difference in genome coverage was likely not sufficient to substantially impact the LD between genotyped SNPs and neighbouring QTL in the sliding 1-Mb windows.

**Table 1:** Descriptive statistics of family-survival phenotypes and number of SNPs located in the 1-Mb windows

	NCCCWA		TLUM	
	Survival (%)	N	Survival (%)	N
Min	26.58	14	2.44	6
Median	76.44	29.50	35	23
Mean	74.90	27.86	38.60	22.15
SD	13.74	6.19	24.11	8.87
Max	100	39	90.24	42

*N* number of SNPs located in the 1-Mb long windows

**Fig. 2:**
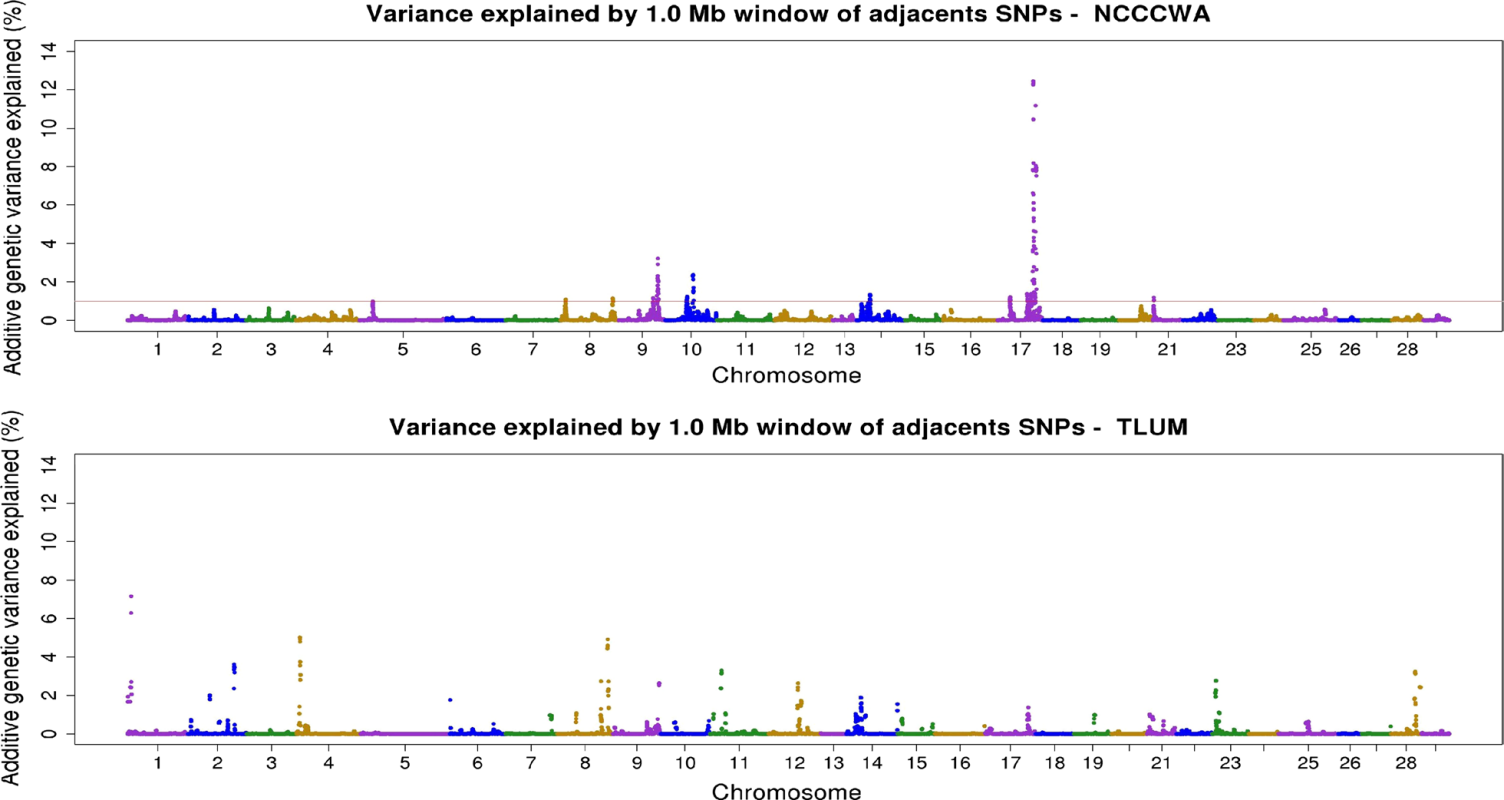
Additive genetic variance for resistance to columnaris disease in two rainbow trout breeding populations explained by 1-Mb sliding SNP windows. The results shown are from iteration 2 of the weighted single-step genome-wide association study (wssGWAS). The year-class 2015 populations are from the USDA-ARS national center for cool and cold water aquaculture (NCCCWA) and from the Troutlodge, Inc. USA May spawning nucleus population (TLUM)

**Fig. 3:**
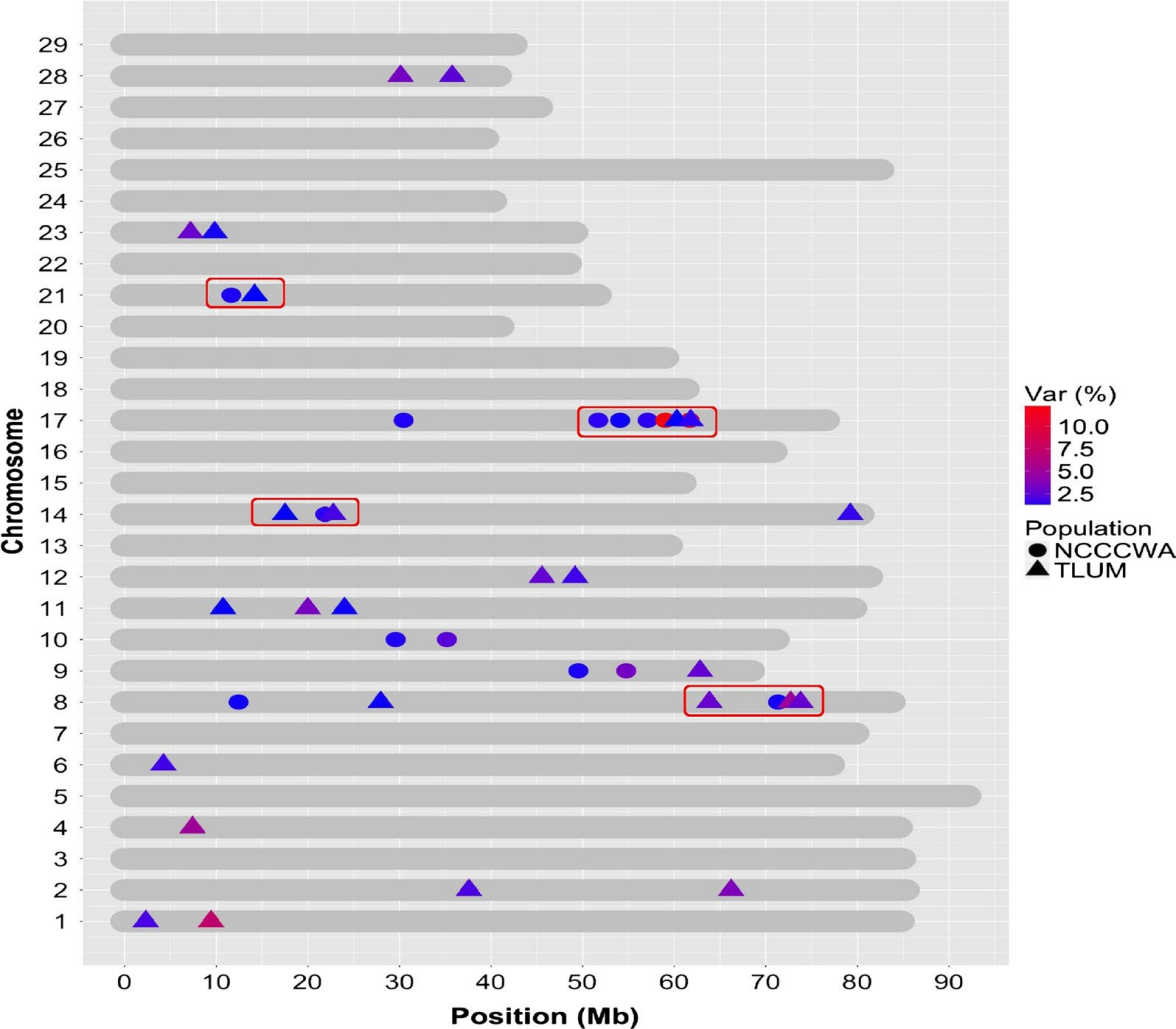
SNP windows that explained more than 1% of the additive genetic variance for resistance to CD in the year-class 2015 populations of TLUM and NCCCWA. Co-localized QTL are marked by a red rectangle

There were 14 windows associated with resistance to CD across six chromosomes in the NCCCWA population, and 26 windows across 13 chromosomes in the TLUM population (Table 2). The percentage of additive genetic variance explained by the SNP windows for each chromosome in each of the two populations is shown in Fig. 2. Only one major QTL (> 10%) was detected in the NCCCWA population, which was revealed by two windows that were located respectively at 59–60 Mb and 61–62 Mb on chromosome Omy 17 (Omy for *Oncorhynchus mykiss*), and explained 12.5 and 11.2% of the genetic variance for resistance to CD, respectively. No major QTL was detected in the TLUM population, but three moderate-effect QTL (≥ 5%) were identified on chromosomes Omy 1, 4, and 8. The genomic distribution and location of windows that were associated with resistance to CD in each population are plotted in Fig. 3. Four QTL regions with co-localized or overlapping windows from both populations were detected on chromosomes Omy 8, 14, 17, and 21 when we used a QTL significance threshold of 1% of the additive genetic variance. Three additional regions of possibly co-localized QTL on Omy 9, 10 and 12 were identified when we used a suggestive threshold of 0.5% of the additive genetic variance (see Additional file 1: Figure S1).

**Table 2:** Chromosomes, regions, and additive genetic variance explained by significant (> 1% of genetic variance) sliding 1-Mb SNP windows for resistance to CD in two rainbow trout aquaculture breeding populations from the National Center for Cool and Cold Water Aquaculture (NCCCWA) and Troutlodge, Inc. (TLUM)

	Omy	Positions (bp)	Var (%)	Start SNP	End SNP
NCCCWA	8	12,432,114–13,425,593	1.10	Affx-88914007	Affx-88948574
	8	71,329,529–72,150,736	1.14	Affx-88923706	Affx-88916609
	9	49,518,971–50,513,124	1.17	Affx-88933418	Affx-88932687
	9	54,751,745–55,726,546	3.22	Affx-88919315	Affx-88950719
	10	29,581,097–30,577,995	1.24	Affx-88920532	Affx-88916735
	10	35,177,214–36,166,546	2.36	Affx-88941227	Affx-88904550
	14	21,882,519–22,880,243	1.34	Affx-88942677	Affx-88914952
	17	30,443,799–31,246,426	1.21	Affx-88915578	Affx-88951951
	17	51,692,366–52,657,204	1.38	Affx-88951951	Affx-88917463
	17	54,077,677–55,070,553	1.07	Affx-88941723	Affx-88930918
	17	57,087,593–58,071,730	1.38	Affx-88928607	Affx-88956489
	17	59,030,557–59,975,343	12.45	Affx-88934715	Affx-88947595
	17	61,685,815–62,683,690	11.17	Affx-88932531	Affx-88933611
	21	11,636,165–12,635,637	1.19	Affx-88924627	Affx-88961166
TLUM	1	2,301,207–3,237,107	1.93	Affx-88920221	Affx-88954173
	1	9,431,533–10,407,809	7.17	Affx-88952286	Affx-88911654
	2	37,586,371–38,445,257	2.00	Affx-88912711	Affx-88941285
	2	66,211,405–67,186,468	3.62	Affx-88959474	Affx-88916549
	4	7,413,629–8,260,785	5.02	Affx-88907515	Affx-88957694
	6	4,230,508–5,197,305	1.76	Affx-88918979	Affx-88930111
	8	27,954,167–28,911,002	1.07	Affx-88910195	Affx-88955224
	8	63,842,515–64,766,638	2.73	Affx-88927151	Affx-88958769
	8	72,702,968–73,532,230	4.92	Affx-88919407	Affx-88912966
	8	73,780,383–74,651,500	2.73	Affx-88940010	Affx-88942756
	9	62,803,755–63,600,917	2.63	Affx-88923649	Affx-88923401
	11	10,741,327–11,703,113	1.04	Affx-88925342	Affx-88919279
	11	19,987,730–20,963,900	3.31	Affx-88942955	Affx-88952300
	11	24,003,333–24,980,010	1.08	Affx-88915068	Affx-88937020
	12	45,554,341–46,523,458	2.63	Affx-88918831	Affx-88918749
	12	49,164,838–50,133,047	1.73	Affx-88916873	Affx-88961655
	14	17,503,583–18,499,177	1.04	Affx-88911639	Affx-88934688
	14	22,775,910–23,775,341	1.90	Affx-88955373	Affx-88908365
	14	79,211,475–79,941,269	1.55	Affx-88904616	Affx-88917061
	17	60,281,825–61,260,250	1.01	Affx-88944127	Affx-88956686
	17	61,789,296–62,742,113	1.37	Affx-88923456	Affx-88919735
	21	14,178,714–15,156,045	1.02	Affx-88954133	Affx-88959956
	23	7,188,750–8,154,673	2.77	Affx-88944584	Affx-88906618
	23	9,817,471–10,809,676	1.13	Affx-88925895	Affx-88930453
	28	30,120,286–31,120,043	3.25	Affx-88955437	Affx-88906826
	28	35,759,045–36,743,773	2.43	Affx-88909402	Affx-88914851

### Comparison of pedigree-based and genomic predictions of breeding values

**Fig. 4:**
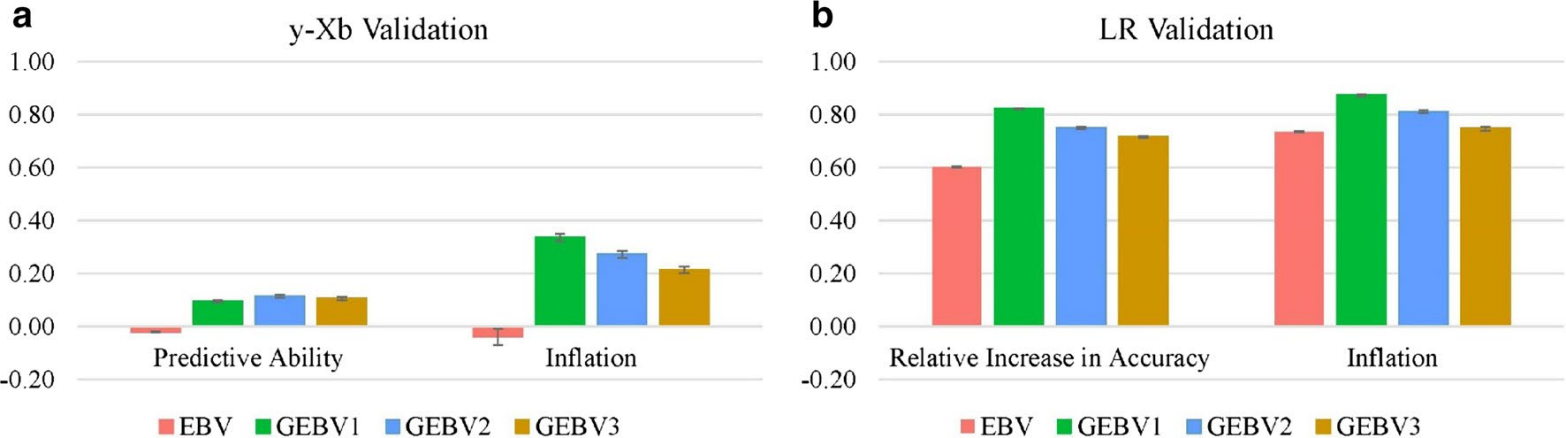
Predictive ability and inflation for validation using adjusted phenotypes (**a**), and relative increase in accuracy and inflation for validation using the LR method (**b**) for the NCCCWA population. EBV is from the traditional evaluation; GEBV1, GEBV2, and GEBV3 are genomic EBV from iterations 1 to 3 of wssGBLUP

**Fig. 5:**
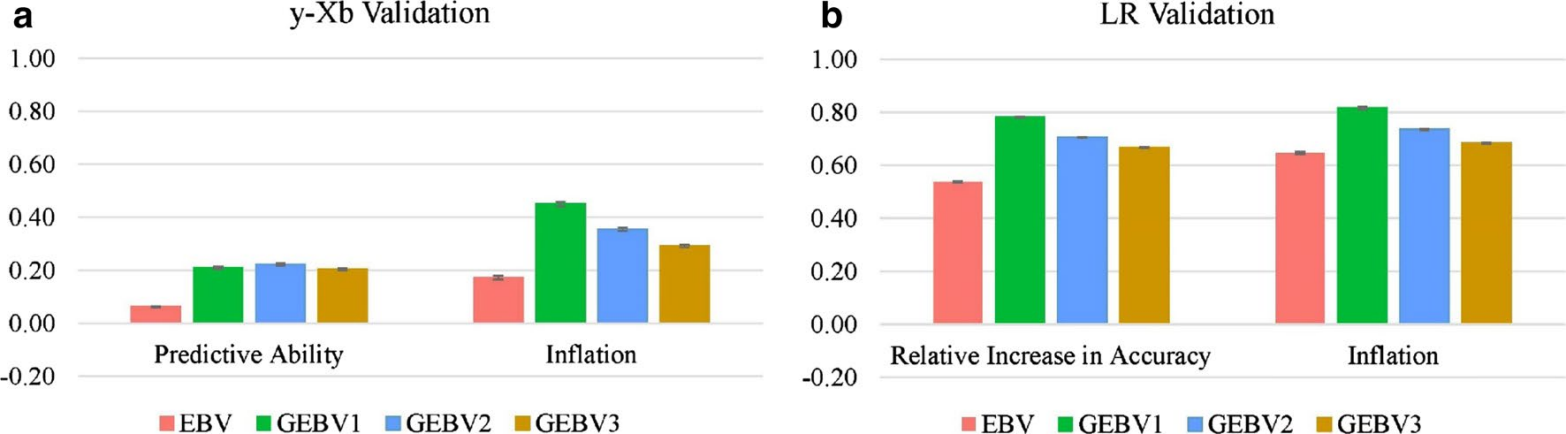
Predictive ability and inflation for validation using adjusted phenotypes (**a**), and relative increase in accuracy and inflation for validation using the LR method (**b**) for the TLUM population. EBV is from the traditional evaluation; GEBV1, GEBV2, and GEBV3 are genomic EBV from iterations 1 to 3 of wssGBLUP

Predictive abilities and inflation for the validation using adjusted phenotypes as benchmarks, and relative increases in accuracy and inflation using (G)EBV as benchmarks are in Figs. 4 and 5 for the NCCCWA and TLUM populations, respectively. For the NCCCWA population, the predictive ability was negative (− 0.02) for EBV, but addition of genomic information increased the predictive ability by up to 0.11 when using the second iteration of weights (GEBV2). Although there was an increase in predictive ability, the predictive abilities were lower than expected for a trait with a heritability between 0.18 and 0.35. For the TLUM population, the predictive ability for EBV and GEBV2 was 0.06 and 0.22, respectively. Both EBV and GEBV were over-dispersed since ${\text{b}}_{1}$ was much lower than 1; the largest values were 0.34 and 0.45 for GEBV1 in the NCCCWA and TLUM populations, respectively.

When the LR validation method was applied, i.e. the complete (G)EBV was used as the benchmark for (G)EBV in each iteration, the relative increase in accuracy from adding phenotypes for selection candidates was greater than 0.5 for EBV and greater than 0.78 for GEBV1 in both populations. Although these values seem to be overestimated, they were close to the accuracy based on PEV, averaged over fold and replication, which were 0.72 and 0.77 for the NCCCWA, and 0.74 and 0.81 for the TLUM populations, for BLUP and ssGBLUP, respectively. Predictive ability and the relative increase in accuracy decreased along the iterations of wssGBLUP, whereas inflation increased. Similar trends have been described by Lourenco et al. [23] as resulting from an excessive shrinkage of SNP weights over iterations, i.e. SNPs that contribute some accuracy are disregarded because they receive a near-zero weight. Less inflated (G)EBV were observed when the benchmark was complete (G)EBV, compared to adjusted phenotypes; values for ${\text{b}}_{1}$ was 0.74 and 0.65 for EBV, and was 0.87 and 0.82 for GEBV1 for the NCCCWA and TLUM populations, respectively.

The overall gain in accuracy by using genomic information to predict breeding values, based on the LR validation, was 37 and 46% for the NCCCWA and TLUM populations, respectively. Based on adjusted phenotypes, the gains in accuracy were larger than 100%; but the magnitude of the predictive ability was very small and EBV were inflated.

## Discussion

### Differences in survival response to columnaris disease challenge

The observed higher survival rate in the CD challenge of families from the NCCCWA, compared to the TLUM population might be the result of the intensive genetic selection for resistance to BCWD applied during the last five generations in the NCCCWA breeding population. The variance components estimated in our recent study [4] indicated that resistance to CD is moderately heritable in both populations (heritability estimates of 0.23 in NCCCWA and 0.34 in TLUM), and that both populations also have a moderate positive genetic correlation between resistance to CD and resistance to BCWD (0.39–0.40). This suggests that both traits might have been genetically improved simultaneously when the NCCCWA population was selected for resistance to BCWD. Conversely, the TLUM population has been under selective breeding pressure for growth, which is likely not genetically correlated with bacterial disease resistance in these aquaculture rainbow trout breeding populations [3, 8]. However, variation in survival rate exists between consecutive challenges even when fish from the same genetic background are evaluated. The higher *F. columnaris* immersion dose (2.08 × 10^8^ CFU for TLUM versus 1.97 × 10^7^ CFU for NCCCWA) might have also contributed to the lower survival rate in the TLUM challenge. However, environmental factors that were expected to improve the survival rate of TLUM fish included age (older by 12 days) and size (bigger) at the start of the disease challenge, and they were also reared at a lower density per tank throughout the challenge.

### Identification of common QTL in two breeding populations

The fact that each population has been under selective pressure for different purposes might have contributed to the detection of different QTL regions for resistance to CD in the two populations. Comparison of the genomic distribution and location of SNP windows associated with resistance to BCWD in the TLUM 2013 year-class from our previous study [24] with the current windows that we identified for resistance to CD in the TLUM 2015 year-class (see Additional file 2: Figure S2), allowed us to identify co-localized QTL on Omy 8, which includes a major QTL for resistance to BCWD and a moderate QTL window for resistance to CD. This supports the notion that selection pressure on one trait might also impact the co-localized QTL for the other trait, and hence selection pressure for resistance to BCWD in previous generations of the NCCCWA population might have affected the allelic distribution of some of the QTL for resistance to CD in that population. A similar comparison of the genome locations of the QTL for resistance to BCWD that were detected among some of the families of the founding generation of the NCCCWA population [24] with those of the QTL for resistance to CD detected in this study after five generations of selective breeding for BCWD resistance is shown in Additional file 3: Figure S3. The two studies share one QTL with a small effect on Omy 10, and also two co-localized or neighbouring QTL on Omy 8. Interestingly, the two QTL on Omy 8 are located near the overlapping QTL for resistance to BCWD and CD in the TLUM population. However, all the other QTL detected for resistance to the two diseases are not shared or co-localized, including the major QTL for resistance to BCWD on Omy 25 and to CD on Omy 17. Considering that the two bacterial species that cause BCWD and CD are both from the *Flavobacterium* genus, it is likely that a certain proportion of common host genes with pleiotropic effects act on the resistance to either pathogen, which might explain the co-localized QTL and the genetic correlation between resistance to these two diseases. However, it is also possible that the genetic correlation is caused in part by physical linkage of QTL that affect each trait separately, as was previously found for resistance to BCWD and spleen size in rainbow trout [25, 26].

Fragomeni et al. [27] showed that the proportion of genetic variance explained by windows and the location of the windows with the largest effect were not consistent across different generations of the same population of a selected commercial broiler line. The fact that the top windows are dynamic in populations under selection and that the variance explained by the windows is not large, suggest that using whole-genome selection instead of region-specific selection would be advantageous in those populations. When windows that explain a certain proportion of variance are identified, a genome-wide selection method that prioritizes or assigns different weights for such windows can help to improve genomic predictions [28], especially in populations with a relatively small number of genotyped animals [23].

It is not surprising that different genome regions are found to affect resistance to CD in the two populations in this study, because bacterial disease resistance is typically a multi-factor trait that is controlled by a complex inheritance system with multiple genes and alternative immunological and physiological pathways, although we cannot exclude the possibility that with better sample size we would have detected more overlapping QTL between the two populations. Of particular interest in this study, are the moderate-major QTL identified on Omy 17 for resistance to CD in the NCCCWA population and the co-localized QTL for resistance to CD and BCWD identified on Omy 8 in the TLUM population.

### Evaluation of the candidate genes for the strongest QTL

We investigated the annotated protein coding genes in RefSeq (NCBI genome annotation of predicted protein coding genes; Accession GCA_002163495.1) for the QTL region with the largest effect on resistance to CD in the NCCCWA population on Omy 17 (59,030,557 bp–59,975,343 bp; Table 2). Overall, 10 coding genes and one pseudogene were identified in this region (see Additional file 4: Table S1), among which only *semaphorin*-*3F*-*like* (regulator of T cell precursor migration and of inflammatory response) might be directly implicated in immune response [29, 30]. However, some of the other proteins may be involved in other important metabolic processes, cell signalling and localisation, maintenance of water homeostasis, or protein synthesis; and hence cannot be overlooked as potentially involved in disease resistance or susceptibility without further investigation. Overall, little is known about the actual function of those proteins in rainbow trout. In addition, we cannot rule out the possibility that other neighbouring genes or sequences that are important for gene expression regulation and in strong LD with the SNPs in that window are the actual causative factor for the strong QTL signal in this region of Omy 17. Clearly, further investigation that is beyond the scope of this report is needed to identify candidate genes for resistance to CD from this QTL region.

### Comparison of pedigree-based and genome-enabled breeding value predictions

In our study, the results obtained for predictive ability and ${\text{b}}_{1}$ from the validation using adjusted phenotypes showed that this is not an appropriate benchmark for binary traits under a threshold model. When this model is applied, the breeding values are estimated on a linear normalized scale, which means that phenotypes or adjusted phenotypes are not on the same scale and do not follow the same distribution as breeding values, reducing the prediction efficiency and increasing bias (i.e., ${\text{b}}_{1}$ much lower than 1). In some other studies [17], the correlation with phenotypes was also divided by the square root of heritability ($h$) to bring it to the accuracy scale. This can produce values that are outside the parameter space (i.e., from 0 to 1). According to Legarra and Reverter [31], even after adjusting phenotypes for fixed effects, a covariance structure across ${\text{y}} - {\text{Xb}}$ can remain, especially when fixed effects are not well estimated, resulting in overestimated accuracy after predictive ability is divided by $h$. In addition, when correlations are divided by $h$ to obtain accuracy, this accuracy is prone to errors if heritability is not well estimated. Although Yoshida et al. [17] reported reasonable values for predictive ability for disease resistance against *Piscirickettsia salmonis* in rainbow trout under ssGBLUP, ${\text{b}}_{1}$ was equal to 0.27, which is close to what we found and is not acceptable under the BLUP assumptions.

One way to circumvent this issue of inappropriate benchmark for binary traits could be to compare family mid-parent (G)EBV on a probability scale with within-family survival probability when progeny of validation fish or selection candidates are challenged with CD pathogens. However, such progeny performance data were not available for the current study. Another way would be to use complete EBV as benchmark for both partial EBV and partial GEBV [32]. However, when the amount of information added from partial to complete data is small, as in our study, complete EBV will be correlated more to partial EBV than to partial GEBV. A third way is to use days to death as a continuous trait instead of a binary mortality trait, which would make comparisons between adjusted phenotypes and (G)EBV more reasonable [5].

Another way to deal with the different scales between phenotypes and (G)EBV is to use an alternative method to assess accuracy and bias/inflation of predictions. We found that the LR method [18] is a promising approach, for binary traits. In the LR approach, both dependent and independent variables are on the same scale because both are (G)EBV that are estimated by the same method using phenotypes. On average, the increase in accuracy from adding phenotypes for validation animals were 0.58, 0.80, 0.73, and 0.70 for EBV, GEBV1, GEBV2, and GEBV3, respectively. The fact that this increase was greater for GEBV than for EBV, means that the partial data predicted the complete data better when genomic information was used. As one of the objectives in animal breeding and genetics is to find models that better predict breeding values and minimize possible changes when phenotypes for selection candidates are included, the LR method may be a reasonable option because it can measure the adequacy of the models. Legarra and Reverter [18] showed that the correlation between complete and partial (G)EBV is a function of accuracies of predictions and reflects the relative increase in accuracy when more phenotypic information is added.

Lourenco et al. [16] reported a small gain of 0.08% in predictive ability by including genomic information in the evaluation for calving ease, a binary trait, in American Angus. This small gain may be due to the result of the small proportion of the genotyped animals having difficult calving (failure) and of the predictive ability being computed as the correlation between a binary phenotype and a normalized estimated breeding value. In a study on heat stress in pigs using reaction norm models, Fragomeni et al. [33] reported a higher accuracy when the benchmark for partial GEBV was the complete GEBV. We observed that inflation and gain in accuracy of predictions from pedigree-based to genomic evaluations can be better assessed when a method is used that allows the comparison of dependent and independent variables on the same scale. This gain in accuracy of predictions was on average 42% in our study, which gives genome-wide selection a great advantage over traditional pedigree-based selection and encourages the application of genomic selection for resistance to CD in rainbow trout breeding populations.

In addition to the LR validation applied in this study, other methods are available to test model fit when using binary data, especially on disease. One method is the odds-ratio (OR) of case–control status and the other is the area under the receiver operating characteristic curve (AUC) [34]. However, the interpretation of results differs among studies. Lee et al. [34] showed in a simulation study that AUC can be used as a measure of model accuracy in case–control studies. Using pregnancy status in a real beef cattle data, Toghiani et al. [35] also applied AUC to evaluate predictive ability of different genomic models. The authors pointed that although high values of AUC indicate better modelling, this measure is not directly comparable to correlations between adjusted phenotypes and GEBV.

### Selection on common QTL versus genomic selection

The use of common or individual QTL for genome-targeted selection in the two populations investigated in this study may not be a promising approach compared to the potential of genome-wide selection. We identified only one moderate-major effect QTL region on chromosome Omy 17 that explains more than 10% of the additive genetic variation in the NCCCWA population and multiple windows with small effects (between 1 and 5%) in both populations. Among those, only four regions that explain more than 1% of the additive genetic variance were common to both populations, which means that a limited amount of the genetic variation can be explained by SNPs associated with QTL that might be useful for genome-target selection. Further validation of the QTL for resistance to CD in more generations from each population is under way, and additional genomic investigation (e.g. genome re-sequencing to discover more SNPs in the QTL regions) could be attempted in the future to help identify potential CD causative variants for resistance to CD and reduce the list of candidate causative genes in the respective QTL regions.

## Conclusions

Columnaris disease in rainbow trout has a complex polygenic inheritance architecture, since it is controlled by several genomic regions that explain a considerable amount of the genetic variance (> 1%). The SNP windows that we found to be associated with resistance to CD do not explain a sufficiently high proportion of genetic variance for choosing genome-targeted instead of genome-wide selection (GS). In addition, because of the small number of overlapping QTL regions between populations, QTL information cannot be used for selection decisions across populations. Genome-wide selection has a greater ability to predict future performance compared to pedigree-based selection. Our analyses also show that correct evaluation of the potential of genomic selection for binary traits requires a proper validation method that accounts for differences in scale when threshold models are used.

## Additional files

**Additional file 1: Figure S1.** SNP windows that explained more than 0.5% of the additive genetic variance for resistance to CD in the year-class 2015 populations of TLUM and NCCCWA. Co-localized QTL are marked by a red rectangle.

**Additional file 2: Figure S2.** SNP windows that explained more than 1% of the additive genetic variance for resistance to BCWD in the TLUM 2013 year-class and CD resistance in the TLUM 2015 year-class. Co-localized QTL are marked by a red rectangle.

**Additional file 3: Figure S3.** SNP windows that explained more than 1% of the additive genetic variance for resistance to BCWD in the NCCCWA 2005 year-class and CD resistance in the NCCCWA 2015 year-class. Co-localized QTL are marked by a red oval.

**Additional file 4: Table S1.** Annotated protein coding genes in RefSeq (NCBI genome annotation of predicted protein coding genes; Accession GCA_002163495.1) found within the QTL region with the largest effect on resistance to CD in the NCCCWA population (Chr. Omy 17; 59,030,557 bp–59,975,343 bp).

## Data Availability

The datasets used and/or analysed during the current study are available upon reasonable request from the corresponding authors.
