# Supplementary figures and images for: Whole-genome mapping of quantitative trait loci and accuracy of genomic predictions for resistance to columnaris disease in two rainbow trout breeding populations
Source: Genet Sel Evol. 2019 Aug 6;51:42. doi: 10.1186/s12711-019-0484-4 (PMC6683352; doi:10.1186/s12711-019-0484-4)

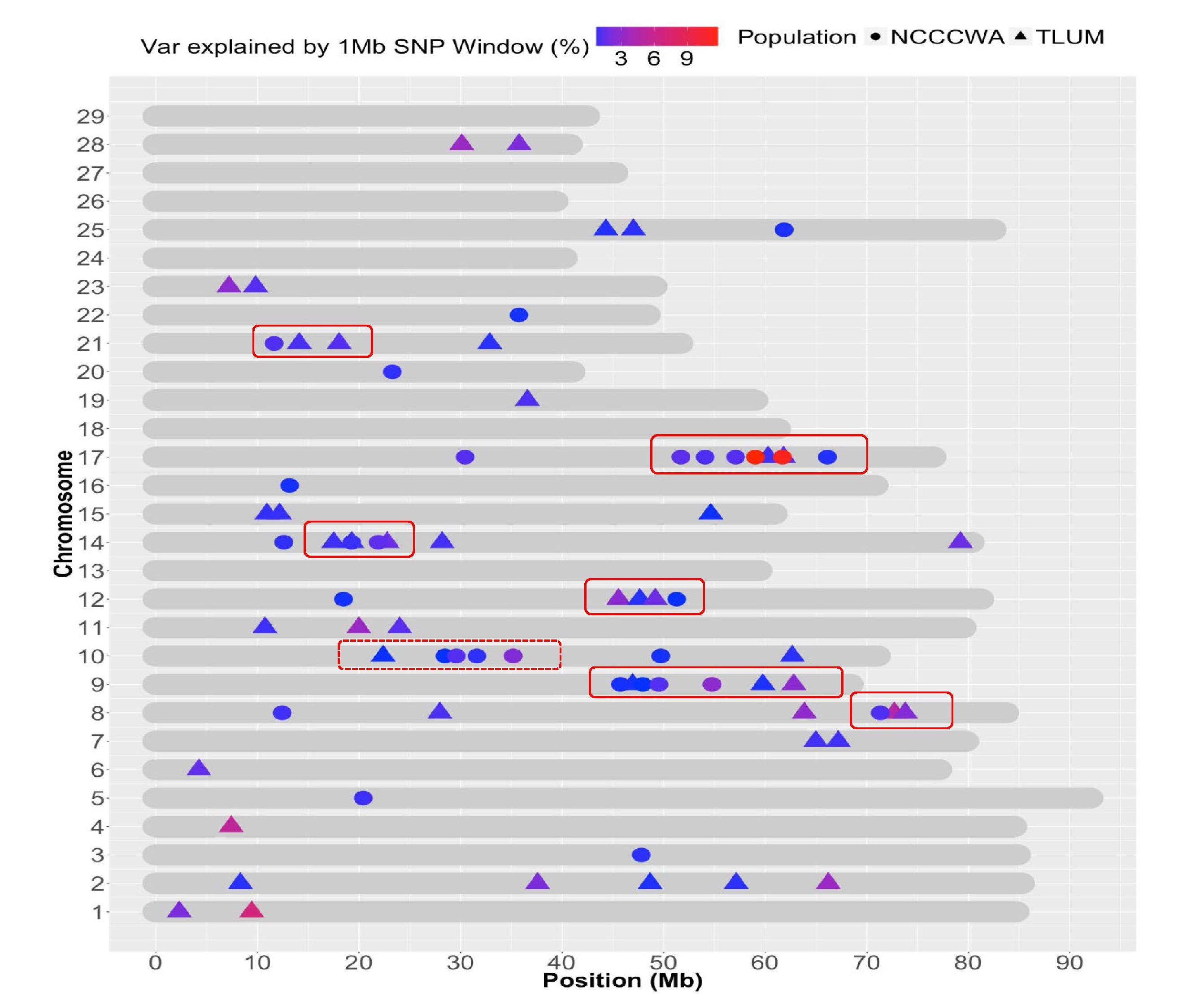

Supplement: Supplementary file 1 — Additional file 1: Figure S1. SNP windows that explained more than 0.5% of the additive genetic variance for resistance to CD in the year-class 2015 populations of TLUM and NCCCWA. Co-localized QTL are marked by a red rectangle. [file 12711_2019_484_MOESM1_ESM.jpg]

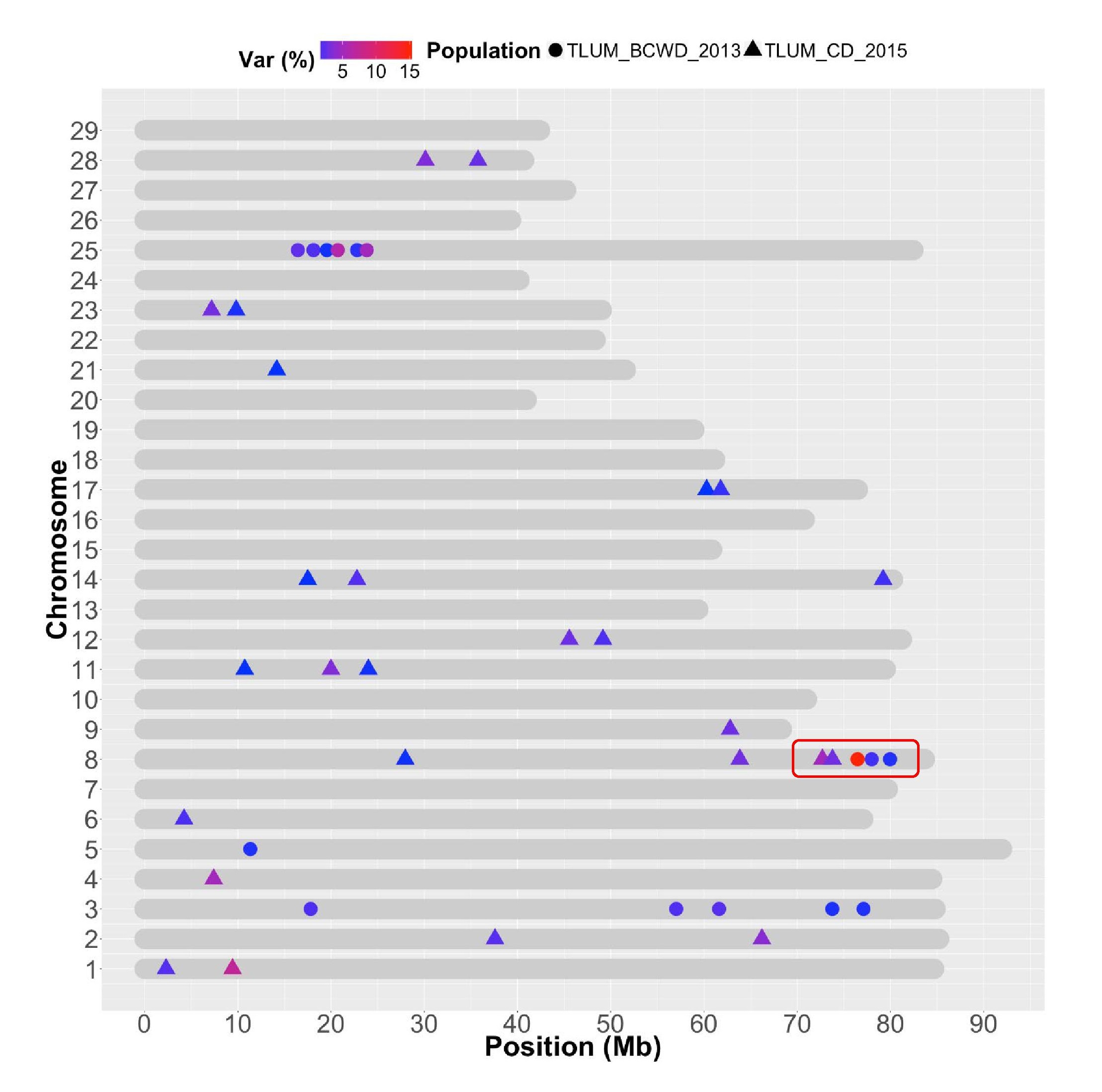

Supplement: Supplementary file 2 — Additional file 2: Figure S2. SNP windows that explained more than 1% of the additive genetic variance for resistance to BCWD in the TLUM 2013 year-class and CD resistance in the TLUM 2015 year-class. Co-localized QTL are marked by a red rectangle. [file 12711_2019_484_MOESM2_ESM.jpg]

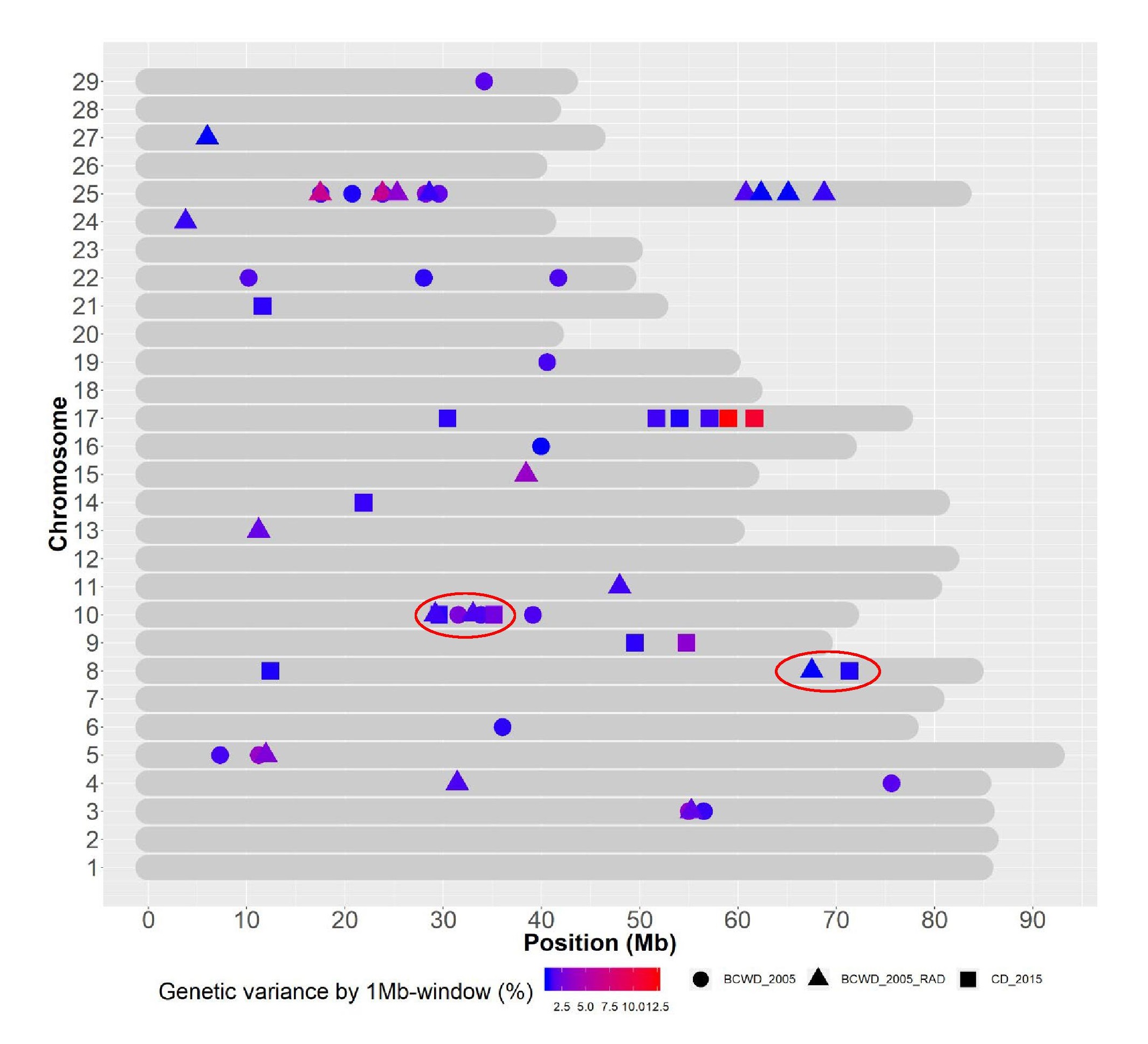

Supplement: Supplementary file 3 — Additional file 3: Figure S3. SNP windows that explained more than 1% of the additive genetic variance for resistance to BCWD in the NCCCWA 2005 year-class and CD resistance in the NCCCWA 2015 year-class. Co-localized QTL are marked by a red oval. [file 12711_2019_484_MOESM3_ESM.jpg]
